# Job Exposure Matrix, a Solution for Retrospective Assessment of Particle Exposure in a Subway Network and Their Long-Term Effects

**DOI:** 10.3390/toxics11100836

**Published:** 2023-10-02

**Authors:** Tesnim Ben Rayana, Pascal Wild, Amélie Debatisse, Valérie Jouannique, Kirushanthi Sakthithasan, Guillaume Suarez, Irina Guseva Canu

**Affiliations:** 1 Center for Primary Care and Public Health (Unisanté), University of Lausanne, 1066 Epalinges-Lausanne, Switzerland; 2 Autonomous Parisian Transportation Administration (RATP), 75012 Paris, France

**Keywords:** particulate matter, long-term exposure, occupational exposure assessment, underground subway, mass concentration, uncertainty

## Abstract

**Highlights:**

**What are the main findings?**

- The job exposure matrix (JEM) provides annual means of PM_10_ concentrations for Parisian subway workers.
- Annual PM_10_ concentrations are estimated over the period of 2004–2020.

**What is the implication of the main finding?**

- The JEM approach is relevant to assess occupational long-term exposure to subway PM_10_.
- The JEM will allow to examen the health effects of long-term exposure to subway PM_10_.

**Abstract:**

Introduction: Health effects after long-term exposure to subway particulate matter (PM) remain unknown due to the lack of individual PM exposure data. This study aimed to apply the job exposure matrix (JEM) approach to retrospectively assess occupational exposure to PM in the Parisian subway. Methods: Job, the line and sector of the transport network, as well as calendar period were four JEM dimensions. For each combination of these dimensions, we generated statistical models to estimate the annual average PM_10_ concentration using data from an exhaustive inventory of the PM measurement campaigns conducted between 2004 and 2020 in the Parisian subway and historical data from the Parisian air pollution monitoring network. The resulting JEM and its exposure estimates were critically examined by experts using the uncertainty analysis framework. Results: The resulting JEM allows for the assignment of the estimated annual PM_10_ concentration to three types of professionals working in the subway: locomotive operators, station agents, and security guards. The estimates’ precision and validity depend on the amount and quality of PM_10_ measurement data used in the job-, line-, and sector-specific models. Models using large amounts of personal exposure measurement data produced rather robust exposure estimates compared to models with lacunary data (i.e., in security guards). The analysis of uncertainty around the exposure estimates allows for the identification of the sources of uncertainty and parameters to be addressed in the future in order to refine and/or improve the JEM. Conclusions: The JEM approach seems relevant for the retrospective exposure assessment of subway workers. When applied to available data on PM_10_, it allows for the estimation of this exposure in locomotive operators and station agents with an acceptable validity. Conversely, for security guards, the current estimates have insufficient validity to recommend their use in an epidemiological study. Therefore, the current JEM should be considered as a valid prototype, which shall be further improved using more robust measurements for some jobs. This JEM can also be further refined by considering additional exposure determinants.

## 1. Introduction

Particulate matter (PM) corresponds to solid or liquid airborne particles with a diameter that spans between 1 µm and 100 µm. Particles with a diameter less than or equal to 10 µm are specified as PM_10_. The literature is abundant and consistently indicates that exposure to ambient PM increases the risk of exacerbation and mortality from cardiovascular and respiratory diseases [1,2,3,4,5], and of the development of cancer [6] and metabolic diseases, such as the diabetes [7].

Studies conducted in the subway systems of several cities showed that subway PM concentrations are frequently higher than ambient PM measured outdoors [8,9,10,11,12,13]. Indeed, in electric subways, PM is primarily produced through three abrasion processes, i.e., between wheel and brake, wheel and rail, and rolling stock and the power supply system [14]. Floor dust particles in tunnels were suggested as a major source of airborne PM in the subway microenvironments [15]. The physical and chemical properties of subway PM differ from ambient PM in terms of size [16] and shape [17], and have an elemental composition rich in heavy metals, mostly the iron [18]. The metallic composition of PM plays a significant role in the induction of oxidative stress and inflammation in experimental studies [19]. Oxidative stress and inflammation are considered as an important pathogenesis mechanism in the development of the chronic diseases induced by exposure to PM [20,21,22]. However, the health effects of PM exposure in the subway network were rarely explored in humans. Studies conducted on subway workers investigated cardiovascular and respiratory outcomes [23,24,25], bronchopulmonary cancer [26], and oxidative stress [27,28]. None of these studies has considered long-term exposure (i.e., longer than two weeks), which would be the most relevant estimate with respect to chronic disease induction [29,30]. This gap in the scientific literature makes it impossible to draw any conclusions about the long-term health effects of subway workers’ occupational exposure to PM [31] and urges further research effort. 

Studies on occupational exposure to PM in subways are quite recent and have not always measured PM_10_. In the Stockholm subway, cleaners and platform workers had the highest PM_10_ exposure, while ticket sellers were the least exposed subway workers, and subway PM concentration was five times higher than at the street level [32]. In the Nanjing subway, occupational exposure to PM in the underground subway workers was also higher compared to that in outdoor workers [33]. However, passengers using urban public transportation of Xi’an, were less exposed to PM_10_ when using subway than when using buses [34]. In the Seoul subway, the mean PM_10_ concentrations on platforms located underground were significantly higher than those at ground level, and PM concentrations at station precincts and platforms exceeded the 24 h acceptable threshold limit regulated by the U.S. Environmental Protection Agency, which is 150 µg/m^3^ [9]. The highest concentration of PM_10_ was reported in transfer pathways of the Seoul subway (83.7 ± 13.8 μg/m^3^) [35]. In the Parisian subway, the highest PM_10_ exposure was observed at the station platforms, followed by the subway cabin and train. PM_10_ levels were negatively correlated with the number of station entrances and correspondence concourses [36]. PM concentrations were below occupational exposure limits (OEL) and varied significantly between jobs. Locomotive operators had the highest PM_10_ exposure (189 μg/m^3^) [37]. 

It is worth mentioning that in France, the OELs for the alveolar deposition fraction of dust (adjusted to an eight-hour work period) and for the total deposition fraction of dust or total dust are set at 0.9 mg/m^3^ and 4 mg/m^3^, respectively. However, the mandatory occupational exposure monitoring only applies to employees whose professional activity directly emits industrial pollution. As subway workers’ exposure to PM is not regulated specifically, there is no systematic occupational exposure monitoring of subway workers. This explains the lack of historical data on PM occupational exposure measurements for subway workers and, consequently, the lack of occupational cohort studies on health effects of subway PM. Nevertheless, the environmental exposure monitoring of PM in the subway networks has been initiated in many countries since the early 2000s. These data could be valuable for approximating occupational exposures retrospectively, providing an appropriate methodology for their use and statistical treatment. 

There are three main approaches used in retrospective exposure assessment: self-reported exposure (mainly through job-specific questionnaires), case-by-case exposure assessment by experts, and the job exposure matrix (JEM). The self-reporting method is advantageous when direct measurements are impractical or unavailable. This approach has several disadvantages, including recall bias, social desirability bias, and lack of specificity. The case-by-case exposure assessment by experts usually offers a greater precision than the self-reporting and JEM method when estimating complex and heterogeneous exposures. However, compared to the latter, the expert assessment is more subjective, costly, time-consuming, and lacks transparency. It can be impossible to apply the case-by-case exposure assessment by experts in a cohort study of 45,000 workers of Parisian transport company. Moreover, in the case of occupational exposure to subway PM, the use of experts could reasonably provide only qualitative exposure assessment (e.g., low, medium, high exposure), especially in a large subway network and two decades backward. Conversely, the use of JEM seems the most promising approach as it enables the quantification of PM levels across different workstations within the network and on a yearly basis. The JEM approach has been increasingly used in occupational epidemiology [38,39]. A JEM is a cross-tabulation of information on jobs and information on exposure to specific workplace hazards [40]. JEMs can be generic (i.e., developed for all jobs in a country) or specific (i.e., developed for jobs of a particular industry or country) [41]. Some company-specific JEMs can be even more detailed, for instance, to specify the exposure at a task-level [42]. Additional dimensions can be incorporated in JEMs to account for temporal and spatial variations in exposure levels, for instance, the calendar period or region [43]. Virtually all types of hazards (e.g., chemical, biological, radiological, ergonomic, or organizational) can be assessed using JEMs [44,45]. Moreover, the JEM approach allows for the consideration of specific physical–chemical properties of the hazard of interest, such as its elemental or isotopic composition [46,47,48]. The exposure assessment in a JEM can be qualitative, semi-quantitative, or quantitative [49,50]. Of course, a precise quantification of the exposure levels through the JEM’s dimensions requires consistent measurement data from important national and international databases [51,52,53,54]. 

With respect to airborne particle exposure, we identified two existent JEMs. The first one is focused on two size fractions of PM in the aluminum industry: PM_2.5_ and total dust, also called total suspended particulates [55]. The second JEM, entitled “MatPUF”, is focused on ultrafine particles (i.e., particles with an average aerodynamic diameter smaller than 0.1 µm) in fifty-seven work processes from multiple industrial sectors [56]. Considering this development, our objective was to evaluate the relevance of a JEM approach for the retrospective assessment of occupational airborne exposure to particles of subway workers. The practical aim of this study was to estimate the historical PM_10_ levels of the workforce of the Parisian subway according to the job held, the workplace as characterized by the metro line and geographic sector within the network, as well as the year in which the job was held. This estimation was based on a large database of exposure measurements that had been assembled [57].

## 2. Materials and Methods

### 2.1. Study Setting

**Figure 1:**
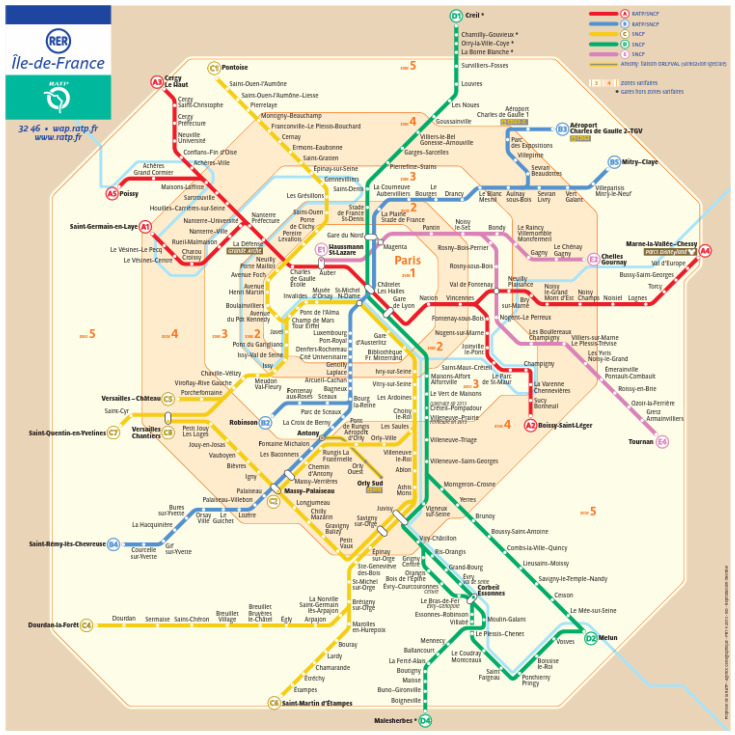
Map of the Parisian railway network: subway and reginal train lines. (Source: https://planparis360.fr/carte/pdf/fr/carte-rer-paris.pdf, accessed on 28 September 2023).

The study was conducted in the Parisian subway network, which is one of the largest public transport networks in the world. It comprises 14 metro lines, among which two are automatized (i.e., line 1 since 2012 and line 14 since its launching) and two intercity lines named the RER lines (Figure 1). All lines are powered by electricity.

### 2.2. Exposure Considered and Measurement Data Used

In this study, we focused on PM_10_ exposure in the subway network, for which we had the largest amount of measurement data available (n = 18,148). These data were centralized in the quantitative database resulting from an exhaustive inventory of PM measurement campaigns and exposure monitoring programs that have been conducted in the company [57]. The quantitative results were abstracted as daily, monthly, or annual average values (in µg/m^3^) along with contextual data abstracted in 30 variables (e.g., the measurement date, the measurement duration and method, the job, and the workplace location). These contextual data allowed for the appropriate management and conversion of different types of data. 

Briefly, we identified three types of PM_10_ measurements (Table 1). The gravimetric method is the reference method in most PM regulations. Its working principle consists of determining the particle mass concentration by weighing a Teflon filter through which ambient air has been pumped at a flowrate of 4 L/min before and after the sampling period [58]. The mass concentration is then obtained by dividing the mass on the filter by the sampled volume (time multiplied by the flow rate) and converted into an occupational exposure based on the sampling duration. The DustTrak (model: DRX, brand: TSI) is a real-time optical measurement device that operates by light scattering using a laser diode [59]. The tapered element oscillating microbalance (TEOM) is another real-time optical measurement device (model: 1400; brand: Thermo Scientific) which operates as a microbalance [60]. This device is validated by the French accreditation committee (COFRAC) and provides PM_10_ concentration averaged over 15 min.

We also identified two types of measurement campaigns: personal campaigns conducted using portable air samplers and stationary campaigns made with fixed samplers placed on railway platforms (Table 1). The personal measurement campaigns included the 2016 Locomotive Operators, the 2017 Security Guards, and the 2019 Work Package 2 (2019 WP2) of the research project ROBoCoP (Respiratory Disease Occupational Biomonitoring Collaborative Project) [61]. The results of this campaign were published previously [36,37,62].

Among the stationary measurement campaigns, we identified the 2016 Mapping campaign conducted in all underground stations of the railway network and the Squales air quality monitoring program set up since 1997 to monitor PM concentration trends at selected stations of the Parisian subway (Chatelet station on line 4 and Franklin Roosevelt on line 1; and Chatelet les Halles, Auber, and Nation stations on the RER A). PM measurement results from this program are available in monthly reports since 2004 (Table 1). Besides these inner-company data on subway PM exposure measurements, we also used data from AirParif, the air quality observatory in the Parisian region. AirParif records annual PM concentrations in the urban background sites of Paris and its suburbs [63]. 

### 2.3. JEM Dimensions

Job is the primary dimension of a JEM. In this study, we considered three types of occupations that comprise most of the underground working population in the Parisian subway (n = 15,000): locomotive operators, security guards, and station agents. The principle of JEM construction involves the definition of homogenous exposure groups (i.e., groups that combine workers with similar exposure level because of tasks or similar work conditions). From the previous studies, we identified that PM concentrations vary depending on the calendar period, line, and geographic sector of the subway network [15,36,57]. Therefore, adding these determinants as additional dimensions to define homogeneous exposure groups in the JEM was important. These dimensions differ across jobs. Locomotive operators are assigned to a specific metro or RER line, except for a small number of reserve locomotive operators who can operate on any line. Each line serves a certain number of aerial (uncovered) or underground stations. Locomotive operators spend most of their work shift inside their cabin, serving all the stations of their assignment line. In addition, they park and unpark trains in underground parking facilities, requiring a short walk through tunnels, and take a break within a few stations along the line.

Security guards are assigned to one of five patrolling geographic sectors (GS) covering Paris and its region. Their work shift begins in each sector from the attachment premises used for instructions and as a cloakroom, then they spend most of their work shift on patrols to maintain security. Their managers provide them with their daily itinerary, in which five and a half hours are spent patrolling, while two and a half hours are spent within the attachment premises, totaling eight hours for a work shift. About 65% of the patrolling time is spent inside the railway network (i.e., metro and RER stations), whereas 35% is spent outside the rail network (i.e., outdoor environment or on buses, tramways, and other vehicles).

Station agents are assigned to a sector that covers a few stations within a given line. They spend most of their work shift at an information desk providing customer service. Station agents may also have to conduct inspection rounds and intervene on ticket vending machines inside the station, thus giving them some mobility, depending on whether they work in a team or independently during their assigned service. 

The staff’s daily work schedule in the Parisian subway is divided into three distinct work shifts: the morning shift, which runs from 5 a.m. to 12 p.m.; the afternoon shift, which runs from 12 p.m. to 7 p.m.; and the evening shift, which runs from 7 p.m. to 2 a.m. For each job, there is a weekly rotation, which leads to an equal distribution of the working shifts between agents (on weekdays or weekends). Therefore, the work shift was not considered as a necessary dimension in the JEM.

### 2.4. JEM Construction

While constructing the JEM, personal measurements were prioritized over stationary measurements [43]. This required some data conversion, computation, and modeling steps, which are described as follows. 

#### 2.4.1. Assessment of the Job-Specific PM_10_ Exposure

To assess the occupational exposure per job within the JEM, we considered the 2019 WP2 campaign as the reference data, as it featured the highest number of personal measurements with the most extended measurement period (i.e., daily work shifts over two weeks of work). Our assumption was that the overall data gathered during this campaign for each job reflects the typical occupational exposure in the context of the 2019 WP2 campaign measurements. Mean concentrations on the three jobs were computed using interval regression to manage the measurements below the limit of detection (LOD) on the log-transformed concentrations of PM_10_. As mentioned earlier, the 2019 WP2 campaign monitored PM_10_ measurements for the three jobs over two work shifts: the morning work shift (i.e., from 5 a.m. to 12 a.m.) for locomotive operators and the afternoon work shift (i.e., from 12 a.m. to 7 pm) for station agents and security guards. Therefore, it was necessary to ensure the comparability between these measurements. For this, we first analyzed the effects of the work shift on raw PM_10_ concentration measured hourly within the Squales campaign at Chatelet station (Table 1) between January 2013 and July 2022. A multiplicative factor between PM_10_ measurements recorded during the morning work shift versus afternoon work shift was then applied to correct the personal mean concentrations in different jobs. 

#### 2.4.2. Integration of the Spatial Dimension

To specify the exposure level according to the job-assignment sites within the JEM, we made use of measurement campaigns with the highest network data coverage for each job. For locomotive operators, logarithmic concentrations of PM_10_ recorded within the 2016 Locomotive Operators campaign were analyzed using a linear interval regression model with the measurement method (i.e., DustTrak or gravimetric) and subway or RER line as independent variables. The aim of this analysis was to predict the ratio of PM_10_ exposure between the different lines and line 7, which was the most exhaustively monitored in the 2019 WP2 campaign. The line-specific mean concentration of the locomotive operators’ exposure for the year 2019 was then estimated by multiplying the locomotive operators’ exposure estimated on line 7 in 2019 (based on the 2019 WP2 campaign data) by the ratio between the locomotive operators’ mean concentrations predicted on each line and the one on line 7 (based on the 2016 Locomotive Operators campaign data) (Supplementary Material S1, Equation (1)). This estimation assumed that the ratio of the predicted locomotive operators’ mean concentrations between the various lines remained the same between 2016 and 2019. It should be noted that no personal measurements were conducted on line 1 within the 2016 Locomotive Operators campaign, as this line was automated in 2012. Therefore, we assessed the associations between the locomotive operators’ exposure to PM_10_ recorded within the 2016 Locomotive Operators campaign and exposure determinants identified in the literature and documented in our database. These determinants were the rolling stock on wheels or iron [64], the train frequency [65], the ventilation rate approximated by the number of fans per line [66], and the depth of the line [36,67]. Exposure to PM_10_ for locomotive operators assigned to line 1 before 2012 was then modeled based on these line parameters.

For security guards, we used the similar strategy applied to the personal measurements data from the 2017 Security Guards campaign. Instead of the line, geographic sector was used as independent variable. Again, security guards’ occupational exposure in the different geographic sectors in the year 2019 has been estimated by multiplying the security guards’ occupational exposure estimated on the Paris sector in 2019 by the ratio between the security guards’ mean concentrations predicted on each geographic sector and the one on the sector of Paris (Supplementary Material S1, Equation (2)). Again, we assumed that the ratio of the security guards’ mean concentrations between the different geographic sectors remains constant between 2017 and 2019.

For station agents, we had no personal measurements except from the 2019 WP2 campaign. Therefore, we first validated the DustTrak device’s accuracy in terms of PM_10_ measurements to use the stationary measurement campaign 2016 Mapping. For this, we examined the correlation between PM_10_ measurements recorded simultaneously within the 2016 Mapping campaign by DustTrak and within the Squales campaign by the TEOM. The results of this analysis showed a strongly positive correlation (Pearson ρ = 0.95) and a TEOM to DustTrack at ratio of 2.05. Log-transformed concentrations from the 2016 Mapping campaign were analyzed using a linear model with the time slot of measurements (≤7:30 a.m; 7:30 a.m–7:59 a.m; 8:00 a.m–8:29 a.m; ≥8:30 a.m), season (i.e., fall and summer), and the sector of assignment as independent variables. In this analysis, we estimated the ratio of the various sectors to the sector line 7 North, for which the 2019 WP2 campaign data were available and adjusted to the time slot and the season. Station agents’ occupational exposure in the various sectors in the year 2019 were thus estimated by multiplying the station agents’ exposure estimated for sector line 7 North in 2019 by the ratio between the mean concentrations predicted on each sector and the one on sector line 7 North (based on the 2016 Mapping data) (Supplementary Material S1, Equation (3)). Again, we assumed that the estimated ratios between underground sectors remained constant between 2016 and 2019. While the sector line 7 North is entirely underground, most sectors include both underground and aerial stations. Mean concentrations on each station agent sector have been estimated via the average of the above concentrations estimated on each of its underground stations based on the 2016 Mapping data and the concentration of its aerial stations that corresponds to outdoor PM_10_ concentration recorded at the urban background measurement stations of AirParif [63]. In 2019, this outdoor PM_10_ concentration was of 20 µg/m^3^. Since it was recorded by TEOM, we converted it into a gravimetric concentration by using the ratio $\frac{\mathrm{gravimetric}\;\mathrm{method}}{\mathrm{TEOM}}$ equal to 0.85. This ratio was determined based on the comparison of TEOM and gravimetric method, as well as on the measurements conducted in the station Ledru Rollin, line 8, by the company’s air quality office. 

#### 2.4.3. Integration of the Temporal Dimension 

In this step, we used the records from the campaigns with repeated measurements over time. We assumed that at each workplace, the personal exposure to PM_10_ is directly proportional, over time, to the PM_10_ concentration in the surrounding environment (i.e., the underground metro and RER stations, and the outdoor environment. Therefore, we modeled the PM_10_ concentrations over time for these three types of environments to estimate occupational exposure over time for each job-assignment site. To model PM_10_ concentrations in underground stations, we used the temporal trend of PM_10_ concentrations recorded within the Squales campaign (Table 1). Logarithmic concentrations recorded separately in metro and RER were analyzed using a linear regression with the sampling year and station as independent variables. To extrapolate the temporal trend from this data, we assumed a constant decrease (on a logarithmic scale) in the annual PM_10_ concentration between 2004 and 2018 in both metro and RER lines. We then assumed that this temporal trend was the same for the rest of the underground stations of the Parisian subway network.

For the aerial stations, we used the temporal trend of ambient PM_10_ annual concentrations measured by AirParif between 2004 and 2019. These concentrations decreased from 30 µg/m^3^, recorded between 2003 and 2005, to 20 µg/m^3^, recorded between 2017 and 2019. This trend was then extrapolated while assuming a constant decrease (on a logarithmic scale) in these annual ambient PM_10_ concentrations between 2004 and 2019. Consequently, the temporal trend of personal exposure to PM_10_ of station agents will directly depend on the RER Squales, metro Squales, and AirParif temporal trends. 

Personal exposure to PM_10_ of locomotive operators while driving between underground stations varies proportionally to the Squales PM_10_ concentrations over time. Also, this exposure while driving in aerial stations varies proportionally to the AirParif concentrations over time. Hence, the temporal slope of the locomotive operators’ exposure to PM_10_ was calculated as the average between the temporal slopes of the Squales data temporal slope and the AirParif data, each weighted, respectively, by the proportion of underground and aerial stations within the line of assignment (Supplementary Material S1, Equation (4)).

For each security guard sector, we characterized the surrounding environments that security guards occupy while patrolling inside and outside the railway. We considered that the surrounding environment while patrolling outside the railway network corresponds to the outdoor environment for all geographical sectors. We then estimated the time spent in total in each of the three surrounding environments over a daily work shift (i.e., the underground metro, the underground RER, and the outdoor) for each security guard sector. As for locomotive operators, the personal exposure to PM_10_ of security guards varies over time proportionally to the Squales concentrations over time while operating in an underground environment and proportionally to the AirParif concentrations while operating in an outdoor environment. Hence, the temporal slope of the security guards’ personal exposure to PM_10_ is calculated as the average between the temporal slopes of the metro Squales data, the RER Squales data, and the AirParif data, each weighted, respectively by the proportion of the total daily time spent in different environment (Supplementary Material S1, Equation (5)).

Similarly, the level of station agents’ occupational exposure varies over time proportionally to the Squales PM_10_ concentrations of the given assignment line type (i.e., metro Squales for metro line or RER Squales for RER lines) while operating in the underground and proportionally to the AirParif concentrations while working at aerial stations. Hence, the temporal slope of the station agents’ occupational exposure was calculated as the average between the temporal slopes of the Squales campaign data and AirParif data, each weighted, respectively, by the proportion of underground and aerial stations within the sector of assignment (Supplementary Material S1, Equation (6)).

### 2.5. JEM Uncertainty Assessment

Three meetings were held with a multidisciplinary team of inner- and outer-company experts, including occupational physicians, hygienists, metrology engineers, epidemiologists, and statisticians, to discuss and validate the strategy of JEM and examine the assumptions required at each step of JEM construction. The final meeting was dedicated to the discussion of JEM’s validity and uncertainty analysis regarding JEM’s exposure estimates. The experts examined uncertainties related to the data quality and the assumptions made during the JEM construction. For these uncertainties, the degree of each one and its impact on the exposure estimates were characterized (e.g., negligible, low, moderate, high, or unknown). Furthermore, the expert team suggested corrective measures for the company to mitigate these uncertainties.

## 3. Results

### 3.1. Work Shift Effect on the Occupational Exposure

The analysis of the work shift effects revealed a multiplicative factor of 1.43 between concentrations recorded in Chatelet (line 4) station during the afternoon work shift versus the morning work shift. Thus, locomotive operators’ exposure recorded in the 2019 WP2 campaign during the morning work shift was corrected using this factor into an afternoon work shift exposure. 

### 3.2. Occupational Exposure Based on the Job-Assignment Site

**Figure 2:**
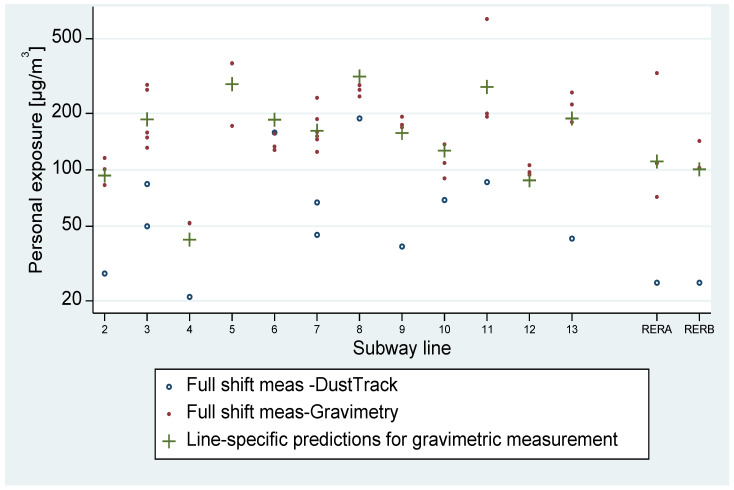
Personal exposure measurements to PM_10_ in the locomotive operators in 2016 according to their assignment line. PM_10_ concentrations are displayed on a logarithmic scale using data extrapolated from the 2016 Locomotive Operators’ campaign.

Exposure measurements to PM_10_ of locomotive operators were distinct according to the subway line (Figure 2). The minimum exposure was reached at line 4 with a value of around 45 µg/m^3^ and reached a six-times-higher value for line 8 with an exposure of around 300 µg/m^3^. Nevertheless, most lines (eight among 14) had PM_10_ values between 100 µg/m^3^ and 200 µg/m^3^.

**Figure 3:**
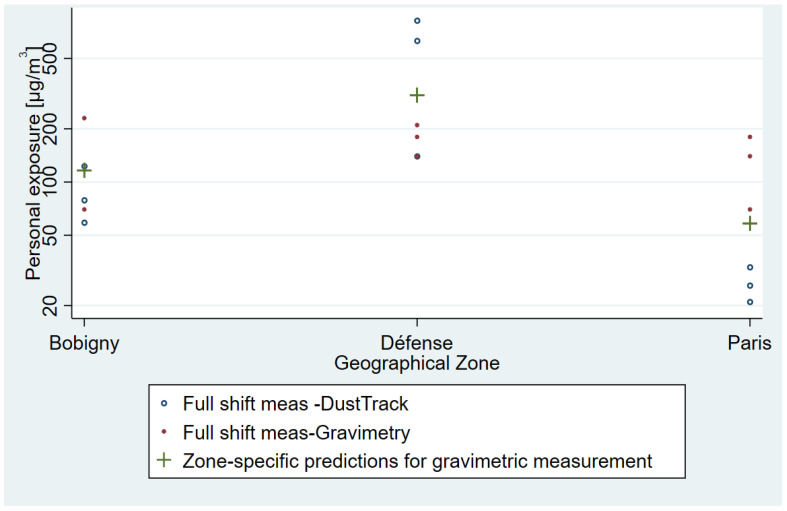
Personal exposure measurements to PM_10_ in the security guards in 2017 according to their assignment sectors. PM_10_ concentrations are displayed on a logarithmic scale using data extrapolated from the 2017 Security Guards campaign.

**Figure 4:**
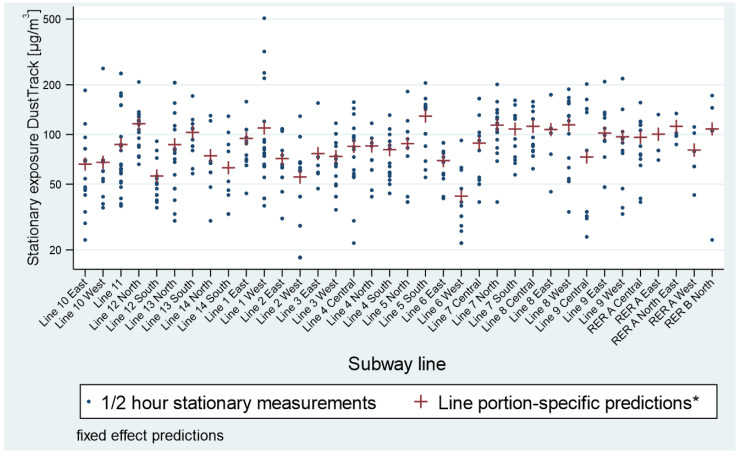
Stationary measurements in the underground sections of the station agent sectors in 2016. PM_10_ concentrations are displayed on a logarithmic scale using data extrapolated from the 2016 Mapping’ campaign.

Exposure measurements to PM_10_ of security guards showed a strong heterogeneity across the three geographic sectors: Paris, La Defense, and Bobigny (Figure 3). Indeed, the geometric mean exposure for the La Defense sector reached about 200 µg/m^3^, nearly twice as high as the one for the Bobigny sector (120 µg/m^3^). The latter was twice as high as the PM_10_ exposure for the Paris sector (about 65 µg/m^3^). For most of the 36 underground portions of the station agent sectors, mean concentrations of PM_10_ ranged between 75 µg/m^3^ and 125 µg/m^3^, which suggests a moderate heterogeneity between these concentrations (Figure 4). Therefore, the hierarchy between PM_10_ concentrations of station agent sectors depends on their aerial station portions. This proportion is equal to or less than 10% on all lines with the exceptions of line 2 and line RER B, which are composed, respectively, of 16% and 84% of aerial stations.

### 3.3. Temporal Trend in the Occupational Exposure

**Figure 5:**
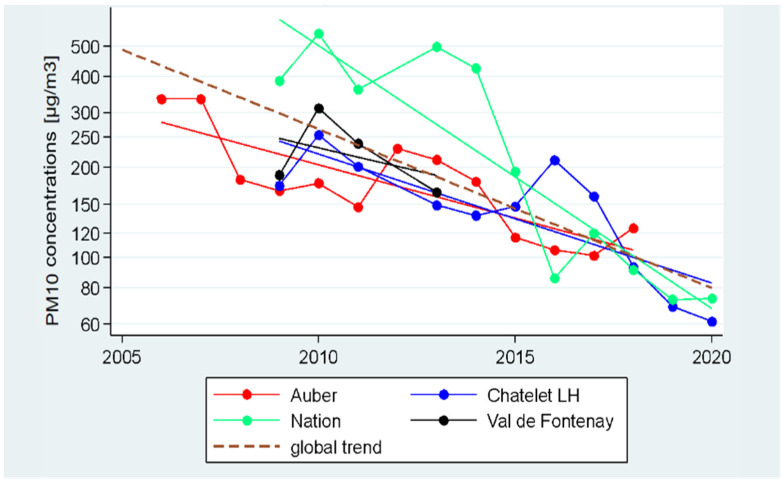
Stationary PM_10_ mass concentrations (in µg/m^3^) recorded between 2006 to 2020 at select underground stations of the intercity line RER A. PM_10_ concentrations are displayed on a logarithmic scale. Solid lines correspond to the trends estimated based on the measurements at the indicated stations, dotted line corresponds to the estimated global trend for the RER.

The concentrations of PM_10_ at the different underground stations of the intercity line RER A showed a global decline between 2006 and 2020 with the same order of magnitude (Figure 5). The global temporal trend, which represents the temporal trend modeled for RER Squales, showed a decrease from nearly 500 µg/m^3^ to 80 µg/m^3^ between 2005 and 2020. This decrease on a logarithmic scale indicates an annual reduction of 12.86%, corresponding to a slope value of −0.05256.

**Figure 6:**
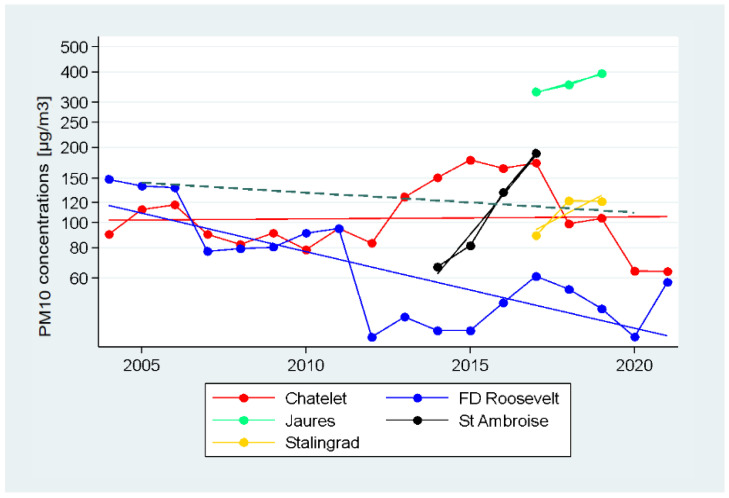
Stationary PM_10_ mass concentrations (in µg/m^3^) recorded between 2004 to 2020 at select underground stations of several metro lines. PM_10_ concentrations are displayed on a logarithmic scale. Solid lines correspond to the trends estimated based on the measurements at the indicated stations, dotted line corresponds to the estimated global trend for the subway.

Chatelet and Franklin Roosevelt metro stations that have been continuously monitored for PM_10_ measurements over more than a decade within the Squales campaign showed a decrease in PM_10_ concentrations with the same order of magnitude (from nearly 85 µg/m^3^ to nearly 60 µg/m^3^) between 2007 and 2020 (Figure 6). However, it should be noted that the variations during certain periods are sometimes different. For example, during the period 2012–2013, PM_10_ concentrations at Chatelet station (line 4) increased, possibly due to the construction work related to the extension of line 4 to the suburbs south of Paris (Mairie de Montrouge station), while those at Franklin Roosevelt station decreased (line 1). The concentrations of PM_10_ at the various underground metro stations showed a weaker global decline over time than the concentrations of PM_10_ at the underground intercity stations previously described. This global trend slope displayed a slight decline going from about 150 µg/m^3^ in 2005 to almost 100 µg/m^3^ in 2020. This decrease on a logarithmic scale indicates an annual reduction of 1.85%, corresponding to a slope value of −0.0079. Outdoor, the AirParif data showed a decreasing trend in PM_10_ concentrations of 2.7% per year on the logarithmic scale, corresponding to a slope value of −0.012.

### 3.4. Exposure Estimates

**Figure 7:**
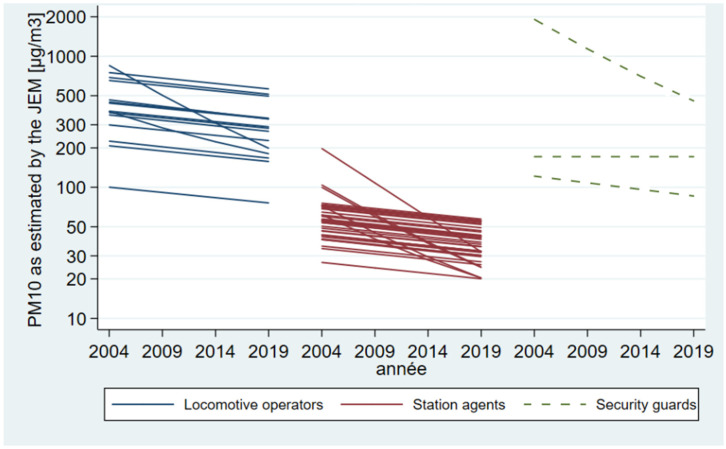
Exposure estimates according to the job, per employment period, in different job assignment sites. Estimates are displayed on a log scale. Dotted lines represent exposure estimates subject to uncertainty.

The constructed quantitative JEM showed that, on average, the annual PM_10_ occupational exposure estimates for locomotive drivers are higher than those for station agents. Moreover, the difference over time in exposure estimates between locomotive operators’ lines is higher than those between station agents’ sectors. We also observed that for both jobs, occupational exposure has decreased differently over time according to the job assignment site (Figure 7). In 2020, the annual PM_10_ exposure estimates between job assignment sites spanned, respectively, from 80 µg/m^3^ to 700 µg/m^3^ for locomotive operators, while for station agents, the estimates were significantly weaker, spanning from 20 µg/m^3^ to 70 µg/m^3^. We provided no quantitative estimates for the two security guards sectors not covered by available measurement data. For the remining three geographic sectors, exposure estimates must be considered with caution due to insufficient quantitative measurements available (Figure 7).

### 3.5. JEM Uncertainties

The uncertainty analysis allowed for the identification of 10 sources of uncertainty that might affect the JEM’s exposure estimates (Supplementary Material Table S1). Among them, only one was considered to have a potentially serious impact on the exposure estimates. It concerned the lack of personal PM_10_ exposure measurements conducted on the security guards. As the experts concluded that the resulting estimates are likely to overestimate the exposure in security guards’ sectors not covered by the measurement companies, this could bias the exposure effect associations in the studies based on the current exposure estimates of security guards. Therefore, we decided to consider these estimates in security guards as provisionally invalid and not to introduce them into the JEM until their improvement based on satisfactory measurement data. 

## 4. Discussion

### 4.1. JEM Relevance for Retrospective Assessment of Occupational Exposure to PM_10_ in Subway Workers

This study demonstrated that the JEM approach is feasible in the context of the occupational exposure assessment in a large subway network. Compared to the other possible approaches of the retrospective exposure assessment, JEM is the only relevant one. Indeed, it would be illogical to ask subway workers to characterize their exposure to PM_10_ at their workplace year per year retrospectively in a self-administered questionnaire or a face-to-face interview since this exposure is difficult to perceive without appropriate measurement. The case-by-case exposure assessment by experts also seems challenging. Indeed, the expert committee involved in this study could only qualitatively describe some (important) changes in work organization, station renovation works, train schedule, rolling stock, and railway maintenance operations, with some insights in the direction of corresponding changes in exposure (i.e., increase or decrease in PM_10_ level). The application of this approach would enable only a rough qualitative assessment of exposure with a rather limited interest for epidemiological application as it disables the assessment of a dose–response relationship. This approach was still applied in some studies, and the results were unconclusive [26,68,69]. In fact, it only allows for the classification of jobs as exposed or not and, at best, for their ordering as a three- or four-class qualitative exposure level. 

Although prospective personal exposure monitoring would have been the best possible way to assess the exposure concentration over time, such a strategy was not actually feasible as it would have been extremely costly. Moreover, it is especially challenging in the context of subway operation. For instance, in the Parisian subway, for security reasons, workers are not allowed to wear any device except their work equipment necessary for their tasks. In the ROBoCoP project, exposure monitoring required two technicians per worker to follow each of them closely during their work shift and wear exposure measurement devices. However, only one technician was accepted in the train cabin because of a lack of space. This increases the study cost and burden on researchers and participants and can only be feasible for a short-term exposure assessment. Consequently, all prospective studies with personal exposure monitoring conducted on subway workers were short-term studies [24,27,68].

The advantage of JEM in the present context lies in its possibility to provide quantitative exposure estimates over a long period retrospectively. It also allows for the utilization of numerous measurement data by their appropriate combination. As documented in our data inventory, each type of data has its own strength, and using them in combination helps strengthen the data informativeness and overall quality of resulting estimates [38]. In this JEM, it was also possible to respect the data hierarchy in terms of their relevance and to favor the personal measurement data as much as possible when estimating occupational exposure [41,70]. Lastly, as recommended for JEM construction, each step of this JEM construction was validated through the cooperation of the occupational physicians and the hygienists assigned to each profession [71]. 

### 4.2. Limitations of the Current JEM

Despite several strengths of this JEM (e.g., quantitative nature, primary personal exposure measurement data used, large temporal coverage, and the inclusion of spatial dimensions), it has some limitations. First, the assumptions on the proportionality of station agents’ exposure with PM_10_ levels recorded on the platforms and on the representativity of the temporal trend recorded in the Squales program of the rest of the lines are strong hypotheses and therefore challenging to confirm. It is also challenging to find data for testing alternative hypotheses. The Squales program gathers data primarily through measurements conducted on lines with rolling stock on wheels and on iron secondarily, and the proportion of both types of lines is nearly equivalent on the whole network. Indeed, it has been shown in different studies, including a recent one focusing on PM concentration measurements conducted over seven years [72], that rolling stock is a determinant of PM concentrations in subway. The second limitation is the incompleteness of some data, particularly data on security guards’ exposure monitoring. Two of the five security guard sectors have not been subjected to measurements, which led to the construction of a JEM that does not cover the exposure of all agents operating in the three jobs. Furthermore, the other three security guard sectors have been monitored insufficiently (during only three work shifts for each one). This increases the uncertainty regarding the validity and precision of the resulting exposure estimates and raises the need of their improvement and validation before their use for epidemiological purposes. Indeed, as all available data with respect to the exposure were used in the estimation of the exposure levels, no independent data were available for validation purposes. Another point worth mentioning is the potential differences exposure between shifts, which is related to the differences according to the time of the day. This could not be accounted for as within the available full shift measurements, this information was not recorded. However, this information does not appear essential because when matching JEM data with job histories, the shifts usually change within jobs and are virtually never available in job histories. 

### 4.3. Further Perspectives

Since the exposure estimates of station agents and locomotive drivers appear sufficiently robust, this JEM can be applied to estimate their annual mean and cumulative exposure to PM_10_ over the period of their employment in the Parisian subway and use these variables in a future epidemiological study. Indeed, these workers constitute a large part (about 70%) of the underground worker population and are part of the occupational cohort EDGAR [73,74]. The final validation of these exposure estimates will be their use in assessing the cumulative exposure of the workers by matching the JEM with their job histories and correlating these individual exposure estimates with the individual health outcomes. Indeed, a positive association between annual mean or cumulative exposure to PM_10_ and cardiovascular or respiratory disease incidence or mortality in these cohorts would confirm the JEM’s predictive validity. 

This JEM should be completed with valid estimates for security guards when more robust data become available. The study has indeed motivated new exposure measurement campaigns targeting this sub-population. The JEM could be further refined with additional exposure determinants, for instance, to distinguish microenvironments or workplaces with different PM_10_ composition. Several studies showed that PM composition varies depending on their sources [9,33,75], and it might be relevant to account for this parameter as well. As this JEM was focused on PM_10_ fraction, future JEMs could include smaller particles, such as PM_2.5_ and ultrafine particles. Although the latter are not yet the subject of regulatory air quality monitoring and are rarely documented, the former are more and more documented based on quantitative personal exposure monitoring [76,77,78,79,80]. 

## 5. Conclusions

This study showed that the JEM approach is relevant for the retrospective assessment of occupational exposure in a large subway network. The resulting JEM focuses on PM_10_ and provides robust exposure estimates for station agents and locomotive operators, who represent a large part of the underground subway workers in Paris. The estimates produced for security guards are uncertain due to the insufficient amount of personal measurement data and should be improved before their use. The validated estimates of exposure can be applied in an epidemiological study to investigate the potential health effects of subway PM_10_. A positive association between annual mean or cumulative exposure to PM_10_ and cardiovascular or respiratory disease incidence or mortality would confirm the predictive validity of this JEM. 

## Figures and Tables

**Table 1:** Summary of PM_10_ measurement data used in this study.

Name of Measurement Campaign	Measurement Type (Location)	Geographical Coverage	Period	Device	Device Time Interval	Measurement Duration	Temporal Coverage	Reported PM Concentration	Number of Recorded Measurements
Squales	Stationary (1 platform)	6 stations (lines 1, 4, 9, 3 on RER A)	January 2004–November 2020	TEOM	15 min	24 h/7 days	Continuous	Daily	7276 369
								Monthly	
						5:30–13:30		Daily	6596 -
								Monthly	
2016 Mapping	Stationary (1 platform)	All network lines and stations	June–December 2016	DustTrak	30 s	30 min	7:30 to 9:30	Average concentration on 30 min (1 platform)	441
Locomotive Operators 2016	Personal (3 locomotive operators per line)	Along all network lines	November–December 2016	Gravimetric	_	=3 work shifts	Morning (=5:00 to 12:00)	Exposure (8 h-TWA)	45
	Personal (1 locomotive operator per line)			DustTrak	30 s	=4 h		Average concentration on = 4 h	14
Security Guards 2017	Personal (each GS team)	3 GS 1, 2, 3 ^†^	January–February 2017, February 2018	Gravimetric	_	3-day work shifts	Afternoon (=12:00 to 19:00)	Exposure (8 h-TWA)	8
				DustTrak	30 s	=4 h		Average concentration on = 4 h	9
WP2 2019	Personal	2 stations of line 7 (station agents)	October 2019	Gravimetric	_	10-day work shifts	Afternoon (=12:00 to 19:00)	Exposure (8 h-TWA)	20
	Personal	Along line 7 (locomotive operators)	October 2019	Gravimetric	_	9-day work shifts	Morning (=5:00 to 12:00)	Exposure (8 h-TWA)	8
	Personal	GS 1 (security guards)	November 2019	Gravimetric	_	9-day work shifts	Afternoon (=12:00 to 19:00)	Exposure (8 h-TWA)	8

^†^ GS, geographic sector: GS1, Paris; GS2, La Défense; and GS3, Bobigny; TEOM: tapered element oscillating microbalance; TWA: time-weighted average; WP2: work package 2 of the ROBoCoP project.

## Data Availability

Restrictions apply to the availability of these data, which were used under license for this study. Data are available from the with the permission of the RATP.

## Supplementary Materials

The following supporting information can be downloaded at: https://www.mdpi.com/article/10.3390/toxics11100836/s1, Table S1: Analysis of the uncertainty resulting from the PM_10_ measurement data gaps and the assumptions considered in the construction of the JEM to PM_10_.

## Abbreviations

AirParif	the air quality observatory in the Parisian region
COFRAC	French accreditation committee (*comité français d’accréditation*, in French)
COPD	chronic obstructive pulmonary disease
GS	geographical sector
LOD	limit of detection
JEM	job exposure matrix
PM	particulate matter
RATP	Parisian subway network (*régie autonome des transports parisiens*, in French)
REA	retrospective exposure assessment
RER	regional express network (*réseau express régional*, in French)
ROBoCoP	respiratory disease occupational biomonitoring collaborative project
TEOM	tapered element oscillating microbalance
TSP	total suspended particulates
UFP	ultrafine particles
WP	work package
