# Supplementary material for: Job Exposure Matrix, a Solution for Retrospective Assessment of Particle Exposure in a Subway Network and Their Long-Term Effects
Source: Toxics. 2023 Oct 2;11(10):836. doi: 10.3390/toxics11100836 (PMC10610788; doi:10.3390/toxics11100836)
Supplement: Supplementary file 1 [file toxics-11-00836-s001.zip › toxics-2611173-supplementary.pdf]

Supplementary material File 1

Equations used to integrate spatial and temporal dimensions into PM10 exposure estimates

Equation (1)

Aim: to predict the ratio of the PM10 exposure between the different lines and line 7 (the most exhaustively monitored in the 2019 WP2 campaign) for locomotive operators

$$[PM_{10}]_{\text{gravimetric, line X, 2019}} = [PM_{10}]_{\text{gravimetric, line 7, 2019 WP2}} * \frac{[PM_{10}]_{\text{line X, 2016 Locomotive Operators}}}{[PM_{10}]_{\text{line 7, 2016 Locomotive Operators}}}$$

Equation (2)

Aim: to predict the ratio of the PM10 exposure between the different geographic sectors of security guards and the geographic sector monitored in the 2019 WP2 campaign

$$[PM_{10}]_{\text{gravimetric, sector X, 2019}} = [PM_{10}]_{\text{gravimetric, sector Paris, 2019 WP2}} * \frac{[PM_{10}]_{\text{sector X, 2017 Security Guards}}}{[PM_{10}]_{\text{sector Paris, 2017 Security Guards}}}$$

Equation (3)

Aim: to predict the ratio of the PM10 exposure between the different sectors and the sector North of the line 7 for station agents

$$[PM_{10}]_{\text{gravimetric, sector X, 2019}}$$

$$= (p_{US}) * [PM_{10}]_{\text{gravimetric, underground sector X, 2019 WP2}} + (1 - p_{US}) * [PM_{10}]_{\text{gravimetric, aerial sector X, 2019 AirParif}}$$

Where p_{US}: proportion of underground stations within the sector of assignment X; (1- p_{US}): proportion of aerial stations within the sector of assignment X.

Equation (4)

Aim: to integrate the temporal dimension in the PM10 exposure prediction for locomotive operators.

$$[PM_{10}]_{\text{gravimetric, line X, year Y}}$$

$$= [PM_{10}]_{\text{gravimetric, line X, 2019}} * \left(\frac{[PM_{10}]_{\text{Squales, year Y}}}{[PM_{10}]_{\text{Squales, 2019}}} * p_{US} + \frac{[PM_{10}]_{\text{AirParif, year Y}}}{[PM_{10}]_{\text{AirParif, 2019}}} * (1 - p_{US})\right)$$

$$= [PM_{10}]_{\text{gravimetric, line X, 2019}} * \left(e^{\text{a Squales (year Y - 2019)}} * p_{US} + e^{\text{a AirParif (year Y - 2019)}} * (1 - p_{US})\right)$$

Where: pUS: proportion of underground stations within the line of assignment X (metro line or RER line); (1-pUS): proportion of aerial stations within the line of assignment X; a Squales: the Squales campaign temporal slope on a log-scale; aAirParif: the AirParif campaign temporal slope on a log-scale

Equation (5)

Aim: to integrate the temporal dimension in the PM10 exposure prediction for security guards.

$$[PM_{10}]_{\text{gravimetric, sector X, year Y}}$$

$$= [PM_{10}]_{\text{gravimetric, sector X, 2019}}$$

$$* \left(\frac{[PM_{10}]_{\text{Squales RER, year Y}}}{[PM_{10}]_{\text{Squales RER, 2019}}} * p_{T\ \text{underground RER}} + \frac{[PM_{10}]_{\text{Squales metro, year Y}}}{[PM_{10}]_{\text{Squales metro, 2019}}} * p_{T\ \text{underground metro}} + \frac{[PM_{10}]_{\text{AirParif, year Y}}}{[PM_{10}]_{\text{AirParif, 2019}}} * p_{T\ \text{outdoor}} \right)$$

$$= [PM_{10}]_{\text{gravimetric, sector X, year Y}}$$

$$* \left(e^{a\ \text{Squales}_{\text{RER}}(\text{year Y - 2019})} * p_{T\ \text{underground RER}} + e^{a\ \text{Squales}_{\text{metro}}(\text{year Y - 2019})} * p_{T\ \text{underground metro}} + e^{a\ \text{AirParif}\ (\text{year Y - 2019})} * p_{T\ \text{outdoor}} \right)$$

where pT: proportion of time spent while on duty in one of the three types of surrounding environments (the underground RER; the underground metro and the outdoor); aSquales: the Squales campaign temporal slope on a log- scale; aAirParif: the AirParif campaign temporal slope on a log-scale.

Equation (6)

Aim: to integrate the temporal dimension in the PM10 exposure prediction for station agents.

$$[PM_{10}]_{\text{gravimetric, sector X, year Y}}$$

$$= [PM_{10}]_{\text{gravimetric, sector X, 2019}} * \left(\frac{[PM_{10}]_{\text{Squales, year Y}}}{[PM_{10}]_{\text{Squales, 2019}}} * p_{US} + \frac{[PM_{10}]_{\text{AirParif, year Y}}}{[PM_{10}]_{\text{AirParif, 2019}}} * (1- p_{US}) \right)$$

$$= [PM_{10}]_{\text{gravimetric, sector X, 2019}} * \left(e^{a\ \text{Squales}\ (\text{year Y - 2019})} * p_{US} + e^{a\ \text{AirParif}\ (\text{year Y - 2019})} * (1- p_{US}) \right)$$

Where pUS : proportion of underground stations within the line of assignment X (metro line or RER line); (1- pUS): proportion of aerial stations within the line of assignment X; aSquales: the Squales campaign temporal slope on a log-scale (i.e., metroSquales as the temporal slope for metro Squales and RERSquales as the temporal slope for 'RER Squales'); aAirParif: the AirParif campaign temporal slope on a log-scale.
